# Hepatocholangioenterostomy

**Published:** 1914-04

**Authors:** 


HEPATOCHOLANGIOENTEROSTOMY· Anschuetz (Abst. in *Zentralblatt f. Chir.* 36, 1913, 1408) reports a case in which, at an operation for gall stones, the hepatic duct was injured and re-united with an apparently perfect result, but after some months, severe icterus with complete retention of bile set in; and at the second operation the liver was found much swollen and full of connective tissue, the region of the hepato-duodenal ligament being transformed into a cicatricial mass in which no connection between the liver and the duodenum could be found. The hepatic duct was represented by a cavity in the liver filled with gall stones; on puncturing the liver in this region clear bile escaped. As it was impossible to unite the duodenum with the portal fissure, a hole was bored in the liver with a Paquelin cautery and this was united with a loop of intestine, the branches of which were united by a button. The suture between the liver and the intestines was strengthened with omentum. The result was decidedly favorable; the icterus disappeared gradually, though not completely. Two and a half years later the patient was feeling fine and, although a slight jaundice persisted, the stools had their normal color.

